# Influence of Terpene Type on the Release from an O/W Nanoemulsion: Experimental and Theoretical Studies

**DOI:** 10.3390/molecules25122747

**Published:** 2020-06-13

**Authors:** Małgorzata Miastkowska, Paweł Śliwa

**Affiliations:** Faculty of Chemical Engineering and Technology, Cracow University of Technology, Warszawska St 24, 31-155 Kraków, Poland; pawel.sliwa@pk.edu.pl

**Keywords:** perillyl alcohol, forskolin, ursolic acid, nanoemulsion, release profile, kinetic analysis, molecular dynamic simulation

## Abstract

The interaction between a drug molecule and its carrier’s components is an important factor which influences the drug release profile. For this purpose, molecular dynamics (MD) may be the in silico tool which can help to understand the mechanism of drug loading/release. The aim of this work is to explain the effect of interactions between different types of terpenes, namely perillyl alcohol, forskolin, ursolic acid, and the nanoemulsion droplet core, on the release by means of experimental and theoretical studies. The basic nanoemulsion was composed of caprylic/capric triglyceride as the oil phase, polysorbate 80 as the emulsifier, and water. The in vitro release tests from a terpene-loaded nanoemulsion were carried out to determine the release profiles. The behavior of terpenoids in the nanoemulsion was also theoretically investigated using the molecular dynamics method. The forskolin-loaded nanoemulsion showed the highest percentage of drug release (almost 80% *w*/*w*) in contrast to ursolic acid and perillyl alcohol-loaded nanoemulsions (about 53% *w*/*w* and 19% *w*/*w*, respectively). The results confirmed that the kinetic model of release was terpene-type dependent. The zero-order model was the best to describe the ursolic acid release profile, while the forskolin and the perillyl alcohol followed a first-order and Higuchi model, respectively. Molecular dynamics simulations, especially energetical analysis, confirmed that the driving force of terpenes diffusion from nanoemulsion interior was their interaction energy with a surfactant.

## 1. Introduction

There are several factors influencing drug release from the vehicle, such as active ingredient properties (solubility, concentration, logP) and carrier properties (type, composition, viscosity) [1]. Drug–carrier interactions are another important factor dictating its release profiles. Drug molecules may directly interact with carriers, which may be lowering their solubility and delay release [2].

Terpenoids, or terpenes, are the largest group of natural bioactive compounds showing multiple nutraceutical activities. They are used worldwide for the treatment of many diseases due to the exhibition of unique biological and pharmacological effects. Terpenoids show cytotoxic, anti-inflammatory, antimicrobial, and antiviral properties, and they also help in the treatment of cancer and cardiovascular diseases. Depending on their carbon chain structure, terpenoids are divided into monoterpenes, sesquiterpenes, diterpenes, sesterpenes, and triterpenes. Perillyl alcohol (monoterpene), forskolin (diterpene), and ursolic acid (triterpene) belong to this group of active compounds with unique and valuable properties. However, it is worth emphasizing that their bioavailability is limited due to their hydrophobic nature and very poor water-solubility [3,4,5].

Among modern drug delivery systems, nanoemulsions are one of the most promising ones. They provide several benefits, such as increased surface area, improved solubility of hydrophobic therapeutic agents, enhancement of the permeation of drugs into the skin, and protection of the actives against external factors (e.g., hydrolysis and enzymatic degradation). The sustained and controlled drug release allows the reduction in the required number of actives and, in consequence, the elimination of potential side effects [6,7,8].

From the physicochemical point of view, a nanoemulsion is a nanoscale colloidal dispersion of two immiscible phases in an oil-in-water (O/W) or water-in-oil (W/O) configuration. The O/W nanoemulsion acts as a carrier for hydrophobic drugs. Its spherical nanodroplets are built of the non-polar tails of the surfactant molecules, which are directed to the micelle hydrophobic core formed by the oil phase. The polar heads of the surfactants are facing towards aqueous external phase [9,10,11]. Different oil and surfactant types which form the nanoemulsion droplets can influence the drug solubilization as a consequence of the difference in density, viscosity, and polarity. The internal structure of nanoparticles, in particular the distribution of a drug within the core, is a key contributing factor to the drug release profile. When the drug is concentrated on the surface of the nanoparticles, they normally exhibit a large initial burst release, whereas those with uniformly loaded drugs tend to provide a more sustained release with a small burst effect. Therefore, apart from release process conditions, the drug behavior towards micelles is also an important factor that may highly affect the bioavailability, drug absorption, and, consequently, the effective design of the delivery system [12,13].

The studies on a variety of drug–micelles interactions can provide structure–energetic or structure–activity relationships and further help in the development of suitable drug delivery systems. Micelles can encapsulate the hydrophobic drugs in their hydrophobic core and thus increase the bioavailability. The encapsulation of drugs in the core depends on the hydrophobic nature of drugs and morphology of micelles, which, in turn, depends on the composition and packing geometry of surfactant monomers [14]. However, drugs may be solubilized, not only in the hydrophobic core, but also on the interface of the micelles. The predominant location of the drug depends on its hydrophobicity and interactions with the surfactant. Highly non-polar lipophilic components tend to be distributed within the hydrophobic core of the particles, whereas more polar lipophilic components may be incorporated within the amphiphilic shell [15]. The possibility to form the drug–surfactant interaction can be enforced by the hydrophobic effect, which is primarily determined by the hydrophobic surface area of the drug molecule. It can be also enforced by the electrostatic effect, which is primarily determined by the charge associated with the drug molecule as well as the surfactant molecules [16]. Due to the hydrophobic environment of the core of micelles, water-insoluble drugs can easily be solubilized and thus loaded for delivery at the required targets.

Predicting drug dissolution with in vivo methods or the release from nanoformulations with in vitro methods become more widely appreciated. In spite of considerable qualitative works confirming the placement of drugs into micelles, detailed quantitative studies of this physical phenomenon are still necessary. The understanding of the encapsulation process on a molecular-level could be very helpful to design an effective drug carrier. Molecular dynamics (MD) simulation is a very powerful tool to understand a number of biomolecular processes and could help to develop and improve drug delivery systems [17]. MD simulations are particularly valuable in addressing issues that are difficult to explore with laboratory measurements. It may help to predict drug delivery prior experiments and, in consequence, allow minimizing the cost of drug design and development [18]. In the context of carrier-mediated drug release, the MD simulations can provide a detailed description of the mechanism of drug loading/release [19,20].

The aim of this study is to explain the effect of interactions between different kinds of active ingredients (mono-, di-, and tri-terpene) and nanoemulsion droplet core on the release mechanism by using MD simulation, and to compare the results with an in vitro release test.

## 2. Results and Discussion

### 2.1. Droplet Size and Polydispersity Index of Terpene-Loaded Nanoemulsion

The results of the measurements of the mean droplet diameter (Z-Ave) and polydispersity index (PDI) of the basic nanoemulsion (NE) as a carrier of perillyl alcohol (NE-PA), forskolin (NE-F), and ursolic acid (NE-UA) are shown in Table 1.

As shown in Table 1, the incorporation of mono- and di-terpene does not affect significantly the mean droplet size of the basic formulation in contrast to ursolic acid addition. This was probably a consequence of the decrease in the solubility of the oil/surfactant micelles. Ursolic acid is very hydrophobic, as can be seen from logP value or its solubility presented in Section 3.1. Forskolin and perillyl alcohol did not affect the droplet size, i.e., they did not decrease the solubility of micelles because they were less hydrophobic. Probably, in the presence of a hydrophilic compound, the size of the micelle could even decrease.

### 2.2. Release of Terpenes

**Figure 1:**
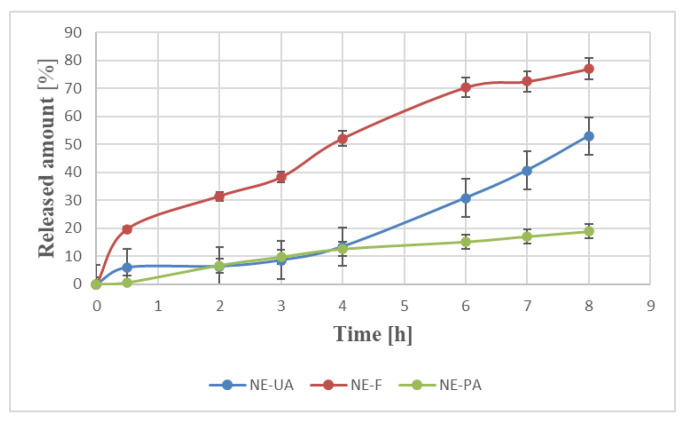
Release profiles of terpenes from the nanoemulsion for perillyl alcohol (NE-PA), forskolin (NE-F), and ursolic acid (NE-UA) respectively, expressed as percentage wt% of released amount (Q (%)) as a function of time. Each value represents the mean ± S.D. (*n* = 3).

In recent years, most studies have focused on the release of one drug from different formulations to select the most effective medium [21,22,23,24,25,26,27]. Here, we have decided to analyze the effect of the drug structure on the release from the same carrier as there is little information concerning that topic. The in vitro release profiles of monoterpene, diterpene, and triterpene from a nanoemulsion are shown in Figure 1.

When it comes to the analysis of the release profiles of terpenes (Figure 1), each of them is released in a controlled and sustained way from a nanocarrier. From the application point of view, it results in the reduction in the dosage and frequency of injection during drug therapy time. These data are consistent with the results of other studies concerning the release of lipophilic drugs from nanoemulsions [22,28]. The forskolin-loaded nanoemulsion showed the highest percentage of drug release (almost 80% *w*/*w*) in contrast with an ursolic acid and perillyl alcohol-loaded nanoemulsion (53% *w*/*w* and 19% *w*/*w*, respectively). This can follow from the lowest lipophilicity of the diterpene (logP = 1.36), among others, and in consequence, higher affinity to the external water phase and then the receptor solution. On the other hand, perillyl alcohol was characterized by the highest water solubility even though it is a more lipophilic compound (logP 1.94). Additionally, monoterpene is characterized by the highest surfactant:oil (S:O) solubility, which may explain higher affinity to micelle oil-surfactant core-shell and, in consequence, the lowest released amount.

Literature reports that highly non-polar lipophilic components tended to be distributed within the hydrophobic core of the particles. On the other hand, more polar lipophilic (like forskolin in our case) components could be incorporated within the amphiphilic shell [15]. The nanoparticles with the drug concentrated on the surface normally exhibited a large initial burst release, whereas those with uniformly loaded drug tended to provide a more sustained release with a small burst effect [12,13].

However, as we mentioned before, there are many factors that affect drug release which make it difficult to explain. The lipophilicity was not the only parameter affecting the drug release. Furthermore, other physicochemical properties of terpenes, such as molar volume, molecular volume, and solubility, should be taken into account, as these parameters correlate with each other. In the case of forskolin, those factors took intermediate values in comparison with the properties of mono- and tri-terpene. Another parameter useful in the prediction of drug transport processes is polar surface area (PSA) [29]. However, forskolin was characterized by the highest value of this factor. Lasoń et al. [30] conducted the release study of selected terpenes (geranic acid, forskolin, ursolic acid) from nanostructured lipid carriers. In this case, the highest final release of the active (nearly 70%) was achieved for ursolic acid. The release profiles of all the tested terpenes were found to be biphasic, with the burst effect at the beginning followed by the gradual release of all the actives.

Thus, drug properties are not the only factors that affect the release. Vehicle properties, vehicle–active interactions, and drug affinity to acceptor solution also play important roles in the prediction of drug release [1]. With regard to drug–vehicle interactions, it is worth noting that the thermodynamic activity of the drug in the formulation is a significant driving force for the release. This driving force reflects the relative activities of the drug in different phases as the drug can be released from the internal phase to the external phase and then to the release medium. [28,31]. This is the reason why we tried to compare in vitro release results with the MD simulation.

### 2.3. Kinetic Analysis

According to the results obtained from the in vitro studies, the release of mono-, di-, and tri-terpene from the nanoemulsion was kinetically evaluated. For this purpose, the zero-order, first-order, Higuchi, and Korsmeyer–Peppas models were used. The kinetic model parameters, including determination coefficient corresponding to each mathematical model, are shown in Table 2.

The results indicate that ursolic acid followed zero-order kinetics (R^2^ = 0.9076). It means that triterpenoid was released slowly, at a constant rate, regardless of the initial drug concentration in the vehicle. A steady amount of the released substances over time can minimize potential side effects because of the reduction in the frequency of drug administration. When it comes to forskolin release profile, the first-order model (R^2^ = 0.9812) was the best to describe the release mechanism of diterpene from the nanoemulsion. This model, according to literature data, was sometimes used to describe the release of hydrophilic drugs e.g., caffeine release profile from a W/O emulsion. Nevertheless, the first-order model also worked well to describe the kinetics of the release of a less water-soluble substance like lidocaine [32]. This model indicated that the drug release rate was proportional to the amount of active ingredient remaining in the formulation and decreased with time [33]. The mechanism of perillyl alcohol (monoterpene) release could be best explained by the Higuchi model, based on the principles of diffusion as expressed by Fick’s first law. This model suggested a pure diffusion release mechanism of the active substance from a vehicle, with no occurring erosion or swelling of the matrix. It described primarily the release of a drug from topical dosage forms, such as hydrogels, creams, and ointments. There were also reports to be found in the literature about lipophilic drug release from nanoformulations according to the Higuchi model [34,35,36,37]. To sum up, the best choice for a kinetic model is a drug-type dependent one. Additionally, Yew and Misran [32] studied the release of different types of drugs (ascorbic acid, caffeine, and lidocaine) from a W/O microemulsion stabilized by the mixture of Span 80 and Tween 80. In the case of their study, the Higuchi model provided the best fit for all active ingredients. Nevertheless, as the hydrophobicity of the active ingredients gradually increased, the release profiles could also be described reasonably well using the first-order model, especially in the case of lidocaine.

### 2.4. Molecular Dynamic Simulations

**Figure 2:**
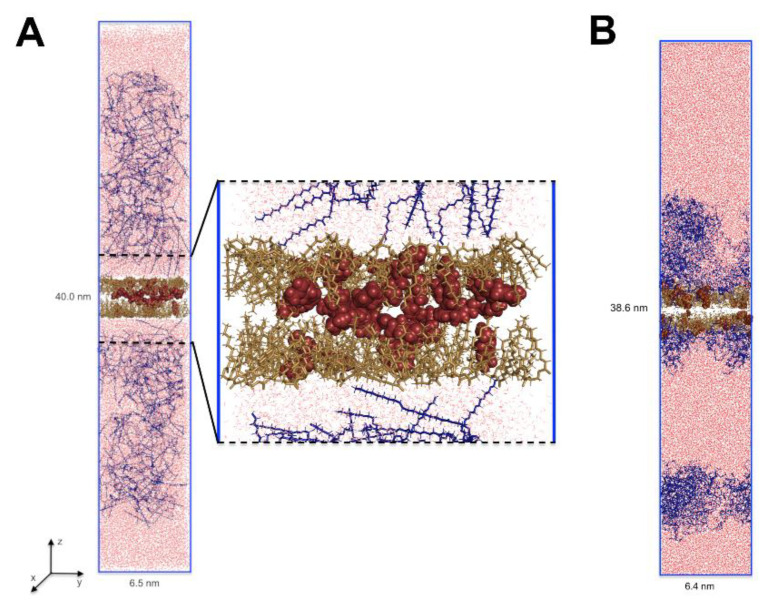
The initial (**A**) and the final (**B**) configuration of the PA/MCT/PS80/water system of MD simulation (x length = y length). The perillyl alcohol is marked as red spheres, caprylic/capric triglyceride (MCT) as sand sticks, polysorbate 80 (PS80) as dark-blue sticks, and water molecules are shown as red lines.

**Figure 3:**
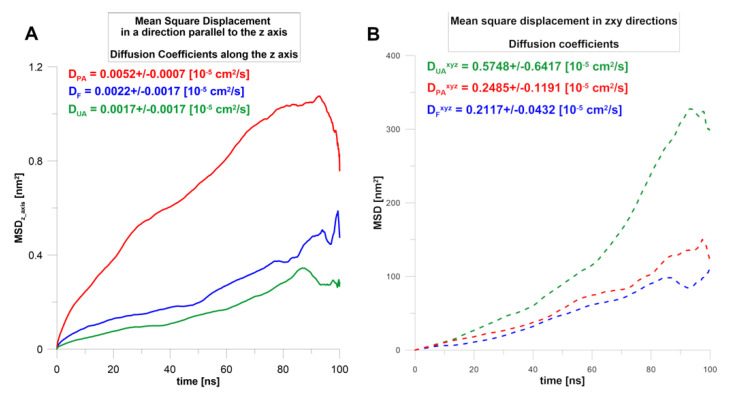
The mean square displacement (MSD) during the 100 ns MD simulations calculated in the *z*-axis direction (**A**) or in three dimensions (**B**). Estimated diffusion coefficients of terpenes. The notation of MSD directions is the same as the coordinates of the simulation box in Figure 2.

To evaluate the diffusion of terpenoids in a nanoemulsion on a molecular level, the series of 100 ns molecular dynamics simulations were performed. For example, the initial and the final configuration for nanoemulsion with perillyl alcohol is shown in Figure 2. This allowed the analysis of the characteristics of the diffusion of active molecules from the oil droplets to the surfactant phase and the analysis of interactions between nanoemulsion components. Generally, for all terpenes, the system in which they were dissolved in the oil phase core (simulation starting point) was not a thermodynamically stable state. The mobility of the molecules in the direction perpendicular to the phase interface, i.e., in the direction desired to release the actives (see the axis designation on Figure 2), was the highest for perillyl alcohol (PA) and the lowest for ursolic acid (UA). The forskolin (F) was moving similarly to UA (Figure 3A). It appears that, at the first stage, the diffusion in the *z*-axis direction depends mainly on the molar mass (Table 3). Additionally, in the case of PA, the MSD(t) plot had two parts: up to 80 ns, the particles changed their positions quite quickly and then significantly decelerated.

**Figure 4:**
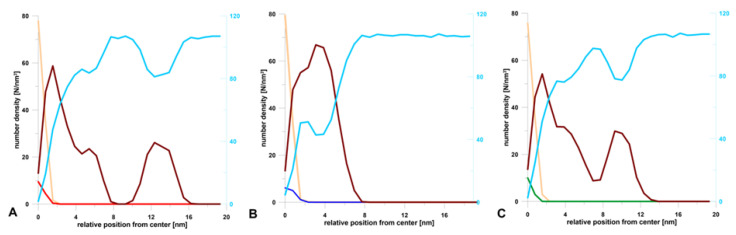
The calculated number densities of water (sky blue), oil (sand), surfactant (dark red), (**A**) perillyl alcohol (red), (**B**) forskolin (blue), and (**C**) ursolic acid (green) for the final configuration of systems after 100 ns MD simulation.

During the simulation, the terpenes were able to diffuse in all directions. The plots of MSD(t) dependence (Figure 3B) show that the terpenes move mainly in the oil phase (during 100 ns), and their global diffusion coefficients are on average 100 times higher compared to those calculated in the *z*-axis direction. The results indicated that UA was the most mobile, while for F and PA, the change of MSD(t) was comparable (Figure 3B). This means that the most hydrophobic compound (logP_UA_ = 6.58, Table 3) could diffuse quite intensely and freely, however mainly in the oil phase. A similar conclusion can be drawn from the calculated number density for the final frames of simulations (Figure 4), where it can be seen that UA was mostly dissolved in the micelle oil core (Figure 4C).

It is well known that nanoemulsion droplets are generally spherical since they are formed due to the reduction of the interfacial area as a consequence of a small radius and high interfacial tension [38]. The initial size and shape of these micelles is determined by the molecular geometry and packing of the surfactant molecules, i.e., their effective head group and tail group dimensions. The particles in nanoemulsions tend to be spherical because the interfacial tension is relatively high and the particle radius is relatively low. As a result, there is a relatively large Laplace pressure favoring the reduction of the interfacial area. It should be noted here that a sphere has the lowest interfacial area for a given volume of material [9]. Drug incorporation may influence nanoemulsion size, polydispersity index, and, what follows, stability [22,39]. Shultz et al. [40] observed a strong influence of the anticancer drug Paclitaxel (hydrophobic drug) on the morphology of poly- (2-oxazoline)-based micelles. In an aqueous solution, the investigated amphiphilic triblock copolymers partially form wormlike micelles. Drug-incorporated triblock copolymers lead to a morphology switch from partial filomicelles to exclusively spherical micelles. This phenomenon was investigated with atomic force microscopy (AFM), cryo-transmission electron microscopy ((cryo-)TEM), and small-angle neutron scattering (SANS). Here, the MD results indicated also the differences in the morphology of nanoemulsion depending on the terpene dissolved in it (Figure 4). To the best of our knowledge, this is the first report concerning such changes in particle morphology. For diterpene (forskolin, Figure 4B), a drop of oil was coated by a single and wide layer of the surfactant, where several forskolin molecules were located in the hydrophobic interior, but most of them on the interface. In this case, a small water layer was also observed at that phase interface (Figure 4B, blue curve). It seems that this structure of micelles may facilitate the transport of the active substance from its hydrophobic interior. Those data are consistent with the reports of other authors [12,13]. The situation was different for mono- and tri-terpene, where it was possible to form two surfactant shells/coatings. The first one was denser and it closely surrounded the oil droplet. The second coating formed at some distance from the main micelle. For the nanoemulsion with PA, these two layers were separated entirely by the aqueous layer (Figure 2B and Figure 4B). In the case of UA, this division was not so pronounced (Figure 4C) and this may be the potential cause of the largest droplet sizes measured by the dynamic light scattering method.

**Figure 5:**
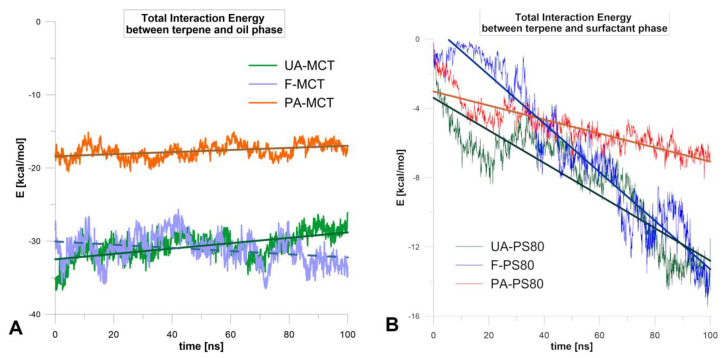
Changes of total interaction energies of terpenoids with oil (**A**) and surfactant (**B**) along with 100 ns MD simulation.

Additional information on the diffusion mechanism was provided by the energetical analysis (Figure 5). The interaction energy of terpenes with the oil phase did not change much during the simulation (SD = 1.0, 2.0, 1.8 kcal/mol, for PA, F, and UA, respectively, Figure 5A). The interaction between triglyceride and di- or tri-terpene was stronger by about 10 kcal/mol than for monoterpene. However, it seems that the driving force of diffusion was the interaction energy with the surfactant (Figure 5B), which increased strongly during the simulation. The largest enhancement was observed for forskolin (Fit lines on Figure 5), which was the most easily released terpene from the nanoemulsion. In contrast, for perillyl alcohol (least released), the smallest enhancement was observed and at the end of the simulation, the interaction energy PA-PS80 was about 10 kcal/mol weaker than for F and UA. This observation correlates well with the experimental studies of terpenes release from a nanoemulsion.

## 3. Materials and Methods

### 3.1. Materials

The nanoemulsion, i.e., the carrier for studied terpenoids, was composed of polysorbate 80 (Caesar&Lorentz GmbH, Hilden, Germany) as emulsifier, caprylic/capric triglyceride (Croda, Krakow, Poland)) as the oil phase, and deionized water. As the active agents, a perillyl alcohol (Aldrich, Poznan, Poland), forskolin (Sabinsa Europe GmbH, Langen, Germany), and ursolic acid (Aldrich) were used. The physical properties of terpenoids are shown in Table 3.

### 3.2. Nanoemulsion Preparation and Characterization

The preparation and characteristics of the nanoemulsion with perillyl alcohol (PA) were described in our previous study [21]. The formulation with forskolin (F) and ursolic acid (UA) was obtained in a similar way. The basic nanoemulsion was composed of caprylic/capric triglyceride as the oil phase, polysorbate 80 as the emulsifier, and water. It was prepared using phase inversion composition (PIC) method by stepwise water addition to the mixture of the oil and surfactant at room temperature (25 °C). The ratio of surfactant to oil (S:O) was 80:20. The basic nanoemulsion was composed of 16% of emulsifier, 4% of oil phase and 80% of water phase. During the preparation of the terpenoid-loaded O/W nanoemulsion, the active substance was incorporated to the mixture of the surfactant and oil and then homogenized with a vortex mixer. The water phase was added, drop by drop, until final formulation was formed. The concentration of perillyl alcohol (PA), forskolin (F) and ursolic acid (UA) in the formulation was related to their maximum solubility in surfactant:oil mixture (Table 3) and reached 0.5%, 0.075% and 0.05% *w*/*w*, respectively. Composition of final formulation was emulsifier (16% *w*/*w*), oil phase (4%), terpene (adequate concentration) and water phase (up to 100% *w*/*w*). Mean droplet diameter and polydispersity index were measured using the dynamic light scattering (DLS) method (Zetasizer Nano ZS, Malvern Instruments, Malvern, UK), at 25 °C.

### 3.3. Release of Terpenoids

The study of the release of a monoterpene-loaded nanoemulsion was described in our previous study [21]. As we mentioned before, the concentration of perillyl alcohol (PA), forskolin (F) and ursolic acid (UA) in the final formulation was related to their maximum solubility in surfactant:oil mixture and reached 0.5%, 0.075% and 0.05% *w*/*w*, respectively. The saturated nanoemulsions were analyzed because saturation is intended to increase the thermodynamic activity which is a significant driving force for the release of the drug. When it comes to composition of receptor solution, it was also dictated by the solubility of the highest possible released amount of each terpene in the receptor solution. For the perillyl alcohol (PA), forskolin (F) and ursolic acid (UA), the mixture of phosphoric buffer (pH = 7.4)/ethanol was combined in ratios of 95/5 *v*/*v*, 70/30 *v*/*v*, 60/40 *v*/*v*, respectively. The release test was carried out using the Spectra/Por Standard regenerated cellulose (RC) membrane, at a temperature of T = 32 °C. Each dialysis bag was filled with a sample of approximately 4 g and placed in a thermostatic chamber containing 250 cm^3^ of receptor solution for 24 h. The concentration of each terpenoid released in the receptor solution was analyzed by means of HPLC (Dionex Ultimate 3000 DAD) equipped with a UV detector and XBridge column (250 mm × 4.6 mm; 3.5 µm) with a precolumn. The mobile phase was isocratic, acetonitrile:water (80:20), and the flow rate was set to 0.5 mL/min. The assay was monitored at a wave-length of 210 nm.

### 3.4. Evaluation of Release Kinetics

The release kinetics was determined by linear regression analysis of the in vitro release curves in four models: zero order (cumulative amount (%) of drug released with time), first-order (log cumulative amount (%) of drug released with time), Higuchi (cumulative amount (%) of drug released with the square root of time), and Korsemeyer–Peppas (log cumulative amount (%) of drug released with log time). The mathematical model that best expressed the kinetic release profile was selected on the basis of the highest coefficient of determination (R^2^) [21,22,23,42,43].

### 3.5. Computational Details

The behavior of terpenoids in the nanoemulsion was also theoretically investigated with a molecular dynamic method. The 3-dimentional structures of the terpenoids (namely, perillyl alcohol (PA), forskolin (F), and ursolic acid (UA)), caprylic/capric triglyceride (the medium chain triglyceride oil, MCT), and polyoxyethylene (20) sorbitan monooleate (non-ionic surfactant, PS80) were prepared in two steps by conformational analysis. First, the InstantJChem tool [44], was used with a Dreiding force field and then the DFT optimization was carried out in Gaussian 16 [45], using CAM-B3LYP functional in combination with 6–31G(d, p) basis set including water as a solvent in a PCM method. The initial system for simulation was prepared using Packmol [46,47] to mimic a pseudo-three-phase system composed of an oil phase with a terpene, surfactant phase, and water phase (TIP3P model). The box dimensions yielded the experimental concentrations and the density of the modelled emulsion about 1000 g/dm^3^. Simulations were carried out in Gromacs 2019.2 [48,49], using periodic boundary conditions in the GAFF force field. They consisted of four steps: minimization of energy, the equilibration: 100 ps isothermally, 100 ps isothermal-isobaric (NPT) and finally, appropriate simulation: 100 ns NPT with 2 fs steps. The simulations were performed in triplicate, and the final results are the arithmetic average of the set of results, while the structures are the result of clustering. Previously, this methodology was successfully applied to study the incorporation of flavonoids into non-ionic surfactant micelles [50,51].

To determine the self-diffusion coefficient (D) of terpenoids, the Einstein relation was used [52]:

(1) $$\underset{t\to \infty }{\lim}❬{\left\|r_{i}\left(t\right)-r_{i}\left(0\right)\right\|}^{2}❭=6\mathrm{Dt}$$

The defined mean square displacement (MSD) and D were calculated by the program, implemented in Gromacs. The MSD was averaged over all molecules and r_i_ was taken as the center of mass positions of the molecules. The diffusion was calculated in one (perpendicular to phase interface) and three dimensions.

## 4. Conclusions

Nanoemulsions are convenient drug delivery systems, especially as hydrophobic as terpenoids. The results confirmed that the kinetic model of release was terpene-type dependent. The zero-order model was the best to describe the ursolic acid release profile, while the forskolin and the perillyl alcohol followed a first-order and Higuchi model, respectively. The experimental results showed that the highest percentage of drug release (almost 80% *w*/*w*) was achieved in the case of diterpene-loaded nanoemulsion, contrary to monoterpene-loaded nanocarrier (about 20%). The molecular dynamics simulations, mainly energetical analysis, confirmed that the driving force of terpenes diffusion from the nanoemulsion interior was their interaction energy with the surfactant. The largest enhancement of interaction energy during the simulation was observed for forskolin, which was the most easily released terpene from a nanoemulsion. Moreover, the simulations revealed that forskolin molecules were located not only in the hydrophobic micelle core but most of them were laid on the interface. It seems that this structure of micelles may facilitate the transport of the active substance from its interior. When the molecular dynamics simulations are compared with the experimentally determined release data, they help us to understand, on a molecular level, why the diterpene was released from the nanoemulsion in the most effective way.

## Figures and Tables

**Table 1:** Properties of the basic and terpene-loaded nanoemulsions.

Sample Name	Terpene	Z-Ave (d. nm) ± S.D./PDI
NE	-	15 ± 1/0.350 ± 0.015
NE-PA	Perillyl alcohol	17 ± 2/0.391± 0.57
NE-F	Forskolin	19 ± 2/0.380 ± 0.027
NE-UA	Ursolic acid	248 ± 15/0.478± 0.089

**Table 2:** The kinetic model parameters fitted to the release results.

Kinetic Model		Parameter	Formulation		
			NE-PA	NE-F	NE-UA
**Zero-order**	$C_{t}=K_{0}\cdot t$	R^2^	0.9535	0.9785	0.9076
		K_0_ (mg/h)	0.432744	0.2304	0.1242
**First-order**	$log(100-C_{t})=$ $\frac{-K_{1}\cdot t}{2,303}$	R^2^	0.965	0.9812	0.8176
		K_1_ (h^−1^)	0.025333	0.1787	0.09189
**Higuchi**	$C_{t}=K_{H}\cdot \sqrt{t}$	R^2^	0.996	0.9655	0.784
		K_H_ (mg/h^1/2^)	1.619123	0.8379	0.4226
**Korsmeyer–Peppas**	$logC_{t}=$ $logK_{KP}+n\cdot logt$	R^2^	0.9396	0.9564	0.7319
		K_HP_ (h^−n^)	1.845	25.287	6.227
		n	1.2444	0.51778	0.807

**Table 3:** Physicochemical properties of studied terpenoids.

Chemical Name	Structure	Molecular Weight (g/mol)	logP ^a^	Water Solubility ^a^ (mg/mL)/logS ^b^ (mol/mL) at pH = 7.4	Polar Surface Area ^b^ (Å^2^)	Molar Volume ^a^ (cm^3^)	S:O Solubility (mg/mL)
Perillyl alcohol	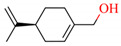	152.2	1.94	1.9/−2.25	20.23	161.8	9
Forskolin	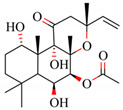	410.5	1.36	1.1/−3.42	113.29	331.3	6
Ursolic acid	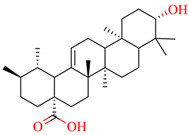	456.7	6.58	0.00059/−4.73	57.53	414.7	2.5

^a^ data taken from PubChem Database [41] or ^b^ calculated using Marvin Sketch.

## References

[1] Eros I., Abu-Eida E.Y., Csóka I., Sánta Z., Cserne A., Kövér T. (2003). Optimization of drug release from dermatological semisolid preparations. Drug Dev. Res..

[2] Zeng L., An L., Wu X. (2011). Modeling Drug-Carrier Interaction in the Drug Release from Nanocarriers. J. Drug Deliv..

[3] Wang G., Tang W., Bidigare R.R. (2005). Terpenoids As Therapeutic Drugs and Pharmaceutical Agents. Natural Products.

[4] Silva M., David J., Silva L., Santos R., David J., Lima L., Reis P., Fontana R. (2012). Bioactive oleanane, lupane and ursane triterpene acid derivatives. Molecules.

[5] Perveen S. (2018). Introductory Chapter: Terpenes and Terpenoids. Terpenes and Terpenoids.

[6] Akhtar N., Verma A., Pathak K. (2017). Exploring preclinical and clinical effectiveness of nanoformulations in the treatment of atopic dermatitis: Safety aspects and patent reviews. Bull. Fac. Pharm. Cairo Univ..

[7] Mishra B.B.T.S., Patel B.B., Tiwari S. (2010). Colloidal nanocarriers: A review on formulation technology, types and applications toward targeted drug delivery. Nanomed. Nanotechnol. Biol. Med..

[8] Lovelyn C., Attama A.A. (2011). Current State of Nanoemulsions in Drug Delivery. J. Biomater. Nanobiotechnol..

[9] McClements D.J. (2012). Nanoemulsions versus microemulsions: Terminology, differences, and similarities. Soft Matter..

[10] Anton N., Vandamme T.F. (2011). Nano-emulsions and micro-emulsions: Clarifications of the critical differences. Pharm. Res..

[11] Miastkowska M., Lasoń E., Sikora E. (2018). Wolińska-Kennard, K. Preparation and Characterization of Water-Based Nano-Perfumes. Nanomater.

[12] Li Y., Wong H.L., Shuhendler A.J., Rauth A.M., Wu X.Y. (2008). Molecular interactions, internal structure and drug release kinetics of rationally developed polymer-lipid hybrid nanoparticles. J. Control. Release.

[13] Yukuyama M.N., Kato E.T., Lobenberg R., Bou-Chacra N.A. (2017). Challenges and Future Prospects of Nanoemulsion as a Drug Delivery System. Curr. Pharm. Des..

[14] Mukhija A., Kishore N. (2018). Drug partitioning in individual and mixed micelles and interaction with protein upon delivery form micellar media. J. Mol. Liq..

[15] McClements D.J. (2011). Edible nanoemulsions: Fabrication, properties, and functional performance. Soft Matter..

[16] Enache M., Volanschi E. (2012). Spectroscopic investigations of the molecular interaction of anticancer drug mitoxantrone with non-ionic surfactant micelles. J. Pharm. Pharm..

[17] Albano J.M.R., de Paula E., Pickholz M. (2018). Molecular Dynamics Simulations to Study Drug Delivery Systems. Molecular Dynamics.

[18] Li Y., Hou T. (2010). Computational Simulation of Drug Delivery at Molecular Level. Curr. Med. Chem..

[19] Katiyar R.S., Jha P.K. (2018). Molecular simulations in drug delivery: Opportunities and challenges. WIREs Comput. Mol. Sci..

[20] Saurabh S., Sivakumar P.M., Perumal V., Khosravi A., Sugumaran A., Prabhawathi V. (2020). Molecular Dynamics Simulations in Drug Discovery and Drug Delivery. A Integrative Nanomedicine for New Therapies.

[21] Miastkowska M., Konieczna M., Lasoń E., Tabaszewska M., Sikora E., Ogonowski J. (2018). The release of perillyl alcohol from the different kind of vehicles. Curr. Pharm. Biotechnol..

[22] Miastkowska M., Sikora E., Ogonowski J., Zielina M., Łudzi A. (2016). The kinetic study of isotretinoin release from nanoemulsion. Colloids Surf. A Physicochem. Eng. Asp..

[23] Miastkowska M., Wójtowicz S. (2020). The kinetic study of dexamethasone release from multiple emulsions. Przem. Chem..

[24] Yener G., Dal Ö., Üner M. (2009). Effect of vehicles on release of meloxicam from various topical formulations. Open Drug Deliv. J..

[25] Özsoy Y., Güngör S., Cevher E. (2004). Vehicle effects on in vitro release of tiaprofenic acid from different topical formulations. IL Farmaco.

[26] Reddy K.R., Sandeep K., Reddy P.S. (2011). In vitro release of ibuprofen from different topical vehicles. Asian J. Pharm. Sci. Res..

[27] Nayak A.S., Jain A., Rathore P., Sumbhate S., Nayak S. (2009). A comparative release study of lisinopril from different vehicles. Int. J. Pharm. Pharm. Sci..

[28] Khalil R.M., Basha M., Kamel R. (2015). Nanoemulsions as parenteral drug delivery systems for a new anticancer benzimidazole derivative: Formulation and in-vitro evaluation. Egypt Pharm. J..

[29] Osterberg T., Norinder U. (2000). Prediction of Polar Surface Area and Drug Transport Processes Using Simple Parameters and PLS Statistics. J. Chem. Inf. Comput. Sci..

[30] Lasoń E., Sikora E., Ogonowski J., Tabaszewska M., Skoczylas Ł. (2016). Release study of selected terpenes from nanostructured lipid carriers. Colloids Surf A Physicochem. Eng. Asp..

[31] Chime S.A., Kenechukwu F.C., Attama A.A. (2014). Nanoemulsions—Advances in Formulation, Characterization and Applications in Drug Delivery. Application of Nanotechnology in Drug Delivery.

[32] Yew H.-C., Misran M.B. (2016). Nonionic Mixed Surfactant Stabilized Water-in-Oil Microemulsions for Active Ingredient In Vitro Sustained Release. J. Surfact. Deterg..

[33] Bruschi M. (2015). Mathematical Models of Drug Release, Strategies to Modify the Drug Release from Pharmaceutical Systems.

[34] Drais H.K., Hussein A.A. (2015). Formulation and characterization of carvedilol nanoemulsion oral liquid dosage form. Int. J. Pharm. Pharm. Sci..

[35] Monteiro L.M., Lione V.F., do Carmo F.A., do Amaral L.H., da Silva J.H., Nasciutti L.E., Rodrigues C.R., Castro H.C., de Sousa V.P., Cabral L.M. (2012). Development and characterization of a new oral dapsone nanoemulsion system: Permeability and in silico bioavailability studies. Int. J. Nanomed..

[36] Pradhan M., Singh D., Murthy S.N., Singh M.R. (2015). Design, characterization and skin permeating potential of Fluocinolone acetonide loaded nanostructured lipid carriers for topical treatment of psoriasis. Steroids.

[37] Borges V.R.A., Simon A., Sena A.R.C., Cabral L.M., de Sousa V.P. (2013). Nanoemulsion containing dapsone for topical administration: A study of in vitro release and epidermal permeation. Int J. Nanomed..

[38] Pavoni L., Perinelli D.R., Bonacucina G., Cespi M., Palmieri G.F. (2020). An Overview of Micro- and Nanoemulsions as Vehicles for Essential Oils: Formulation, Preparation and Stability. Nanomater.

[39] Miastkowska M., Sikora E., Lasoń E., Garcia-Celma M.J., Escribano-Ferrer E., Solans C., Llinas M. (2017). Nano-emulsions as vehicles for topical delivery of forskolin. Acta Biochim. Pol..

[40] Shultz A., Jaksch S., Schubel R., Wegener E., Di Z., Han Y., Meister A., Kressler J., Kabanov A.V., Luxenhofer R. (2014). Drug-Induced Morphology Switch in Drug Delivery Systems Based on Poly(2-oxazoline)s. ACS Nano.

[41] Explore Chemistry. https://pubchem.ncbi.nlm.nih.gov/compound/.

[42] Dash S., Murthy P.N., Nath L., Chowdhury P. (2010). Kinetic modeling on drug release from controlled drug delivery systems. Acta Pol. Pharm..

[43] Costa P., Lobo J.M.S. (2001). Modeling and comparison of dissolution profiles. Eur. J. Pharm. Sci..

[44] ChemAxon (2015). InstantJChem 15.2.16.0. http://www.chemaxon.com.

[45] Frisch M.J., Frisch G.W., Trucks H.B., Schlegel G.E., Scuseria M.A., Robb J.R., Cheeseman G., Scalmani V., Barone G.A., Petersson H. (2016). Gaussian 16, Revision C. 01.

[46] Martinez L., Andrade R., Birgin E.G., Martinez J.M. (2009). Packmol: A Package for Building Initial Configurations for Molecular Dynamics Simulations. J. Comput. Chem..

[47] Martínez J.M., Martínez L. (2003). Packing optimization for automated generation of complex system’s initial configurations for molecular dynamics and docking. J. Comput. Chem..

[48] Berendsen H.J.C., van der Spoel D., van Drunen R. (1995). GROMACS: A message-passing parallel molecular dynamics implementation. Comput. Phys. Commun..

[49] Abraham M.J., Murtola T., Schulz R., Páll S., Smith J.C., Hess B., Lindah E. (2015). Gromacs: High performance molecular simulations through multi-level parallelism from laptops to supercomputers. SoftwareX.

[50] Śliwa P., Śliwa K., Sikora E., Ogonowski J., Oszmiański J., Nowicka P. (2019). Incorporation of bioflavonoids from Bidens tripartite into micelles of non-ionic surfactants-experimental and theoretical studies. Colloids Surf. B Biointerfaces.

[51] Śliwa K., Śliwa P., Sikora E., Ogonowski J., Oszmiański J., Nowicka P. (2019). Application of Polyethylene/Polypropylene Glycol Ethers of Fatty Alcohols for Micelle-Mediated Extraction of Calendula anthodium. J. Surfactants Deterg..

[52] Einstein A. (1905). On the Motion of Small Particles Suspended in a Stationary Liquid. Ann. Phys..

